# Antics of Watches

**Published:** 1892-02

**Authors:** 


### ANTICS OF WATCHES.

Decidedly the watch is a very queer thing. It possesses some unaccountable peculiarities. Some time ago, when there had been a succession of fine displays of the aurora borealis, it was estimated that in a single night in New York the mainsprings of not less than 3,000 watches broke. The estimate it based on actual enquiries.

Fine, sensitive watches are particularly liable to be affected by electrical atmospheric disturbances. During the months of June, July, and August, when these phenomena are most frequent, there are more mainsprings broken than during all the remaining months of the year. They break in a variety of ways, sometimes snapping into as many twenty-seven pieces.

It is a fact that since the use of electric light has become so general a large number of watches, some of them very fine ones, have become magnetized. While in this condition they are useless as timekeepers. This defect used to be considered incurable, and because of it thousands of watches have been thrown away after much money had been spent on them in vain attempts to persuade them to keep good time.

Among the many methods resorted to were washing the parts in garlic juice, refinishing and passing them through the fire. But all these devices were entire failures, or only in part effective.

These are occasions when it is a very serious matter to have your watch magnetized. The captain of an Atlantic steamer, before putting to sea on a recent voyage was invited to inspect an electric dynamo machine and examined its parts closely.

Soon after getting on board the steamer he noticed that the compass became strangely affected when he approached it. Whether he stood on the right or the left or immediately in front of the compass, the needle would invariably point to him. The compass was worse than useless when he came near it. It was dangerous and might wreck the ship.

This phenomenon alarmed and puzzled the captain not a little. At length he recalled his visit to the dynamo machine, and the true solution of the eccentric behavior of the needle flashed upon him. His watch had become magnetized. When he removed it the needle resumed its constancy to the polar star.

Watches frequently get magetized in iron mines or machine shops, where they are incautiously brought near swiftly running belts.

It is a well known fact among horologists that no watch will keep the same time with two people. The cause has not yet been definitely ascertained, but it would seem that in some mysterious way a watch is affected by the temperament of the wearer. The mere physical difference in gait and movement between different people is not sufficient to account for all the variations that have been observed.

